# Association between the platelet-albumin-bilirubin score and all-cause mortality in ICU-admitted heart failure patients: a retrospective cohort analysis and machine learning-based prognostic modeling

**DOI:** 10.3389/fcvm.2025.1622554

**Published:** 2025-10-23

**Authors:** Zhantao Cao, Jian Li, Guanfa Yuan, Jinghua Ren, Jingting Chen, Kailin Zheng, Yunsu Wang, Zhonghui Lin

**Affiliations:** Department of Cardiology, Xiamen TCM Hospital Affiliated to Fujian University of Traditional Chinese Medicine, Xiamen, Fujian, China

**Keywords:** platelet-albumin-bilirubin score, heart failure, all-cause mortality, machine learning, MIMIC-IV database

## Abstract

**Background:**

The platelet-albumin-bilirubin (PALBI) score has shown prognostic value across multiple medical conditions; nevertheless, its effectiveness in forecasting prognoses among severely ill heart failure (HF) patients treated in Intensive Care Unit (ICU) has yet to be fully established. This study explores the relationship between PALBI scores at ICU admission and all-cause mortality in HF patients admitted to the ICU.

**Methods:**

Drawing on records from the MIMIC-IV version 3.1 critical care database, we included ICU-admitted HF patients and calculated their PALBI scores at admission. Kaplan–Meier survival curves and log-rank tests were used to assess differences in overall mortality at 30 and 360 days across the PALBI tertile groups. Cox regression models based on the proportional hazards assumption were utilized to control for possible confounding variables. In addition, predictive models based on machine learning were constructed using PALBI alongside other clinical features to estimate 30-day mortality, with model performance evaluated through the area under the ROC curve (AUC).

**Results:**

A total of 4,318 participants were included in the study cohort (57% male; median age 73 years). The cumulative incidence of all-cause mortality was 24% at 30 days and 44% at 360 days. Individuals in the top PALBI tertile exhibited markedly higher mortality rates compared to those in the lowest tertile (30% vs. 20% at 30 days and 52% vs. 39% at 360 days). Multivariate Cox regression analysis revealed significant associations of elevated PALBI scores with higher mortality risk at both 30 days (HR: 1.36; 95% CI: 1.12–1.64; *p* *=* 0.002) and 360 days (HR: 1.22; 95% CI: 1.03–1.44; *p* *=* 0.019). Machine learning models effectively discriminated patients at risk of 30-day mortality, with the best performance achieved by Ridge regression (AUC = 0.76).

**Conclusion:**

The PALBI score independently predicts 30-day and 360-day all-cause mortality among ICU-admitted HF patients. These findings suggest that the PALBI score has potential utility for risk stratification and for guiding treatment decisions in the intensive care management of HF.

## 1 Introduction

Heart failure (HF) is a multifaceted syndrome marked by impaired cardiac output, which results in reduced blood flow to vital organs and tissues. HF poses a major public health challenge worldwide, currently impacting around 6.7 million adults in the U.S., with estimates suggesting this number could rise to 8.7 million by 2030 (1). Patients with HF frequently present with severe conditions requiring intensive care; approximately 14% of admissions to cardiac intensive care units (CICU) are primarily due to HF, with a subsequent mortality or rehospitalization rate of 52.8% within one year among ICU HF patients (2, 3).

HF often involves multi-organ dysfunction, particularly hepatic impairment. Liver dysfunction in HF occurs through pathophysiological processes such as congestive hepatopathy and acute cardiogenic liver injury. Moreover, liver disease may exacerbate cardiac dysfunction, establishing a bidirectional cardio-hepatic interaction (4). Abnormalities in hepatic markers, including bilirubin and albumin, frequently occur in patients with HF and are associated with adverse prognoses. For example, increased bilirubin concentrations have been recognized as independent indicators of adverse prognosis in individuals with HF (5). Likewise, hypoalbuminemia and thrombocytopenia are more prevalent among HF patients and significantly correlate with increased mortality risk (6, 7). Collectively, these findings underscore the importance of comprehensive hepatic function assessments in evaluating prognostic outcomes in HF.

Recent studies have demonstrated that the albumin-bilirubin (ALBI) score, a measure of liver function, is a powerful predictor of prognosis in ICU-admitted HF patients, with each unit increase in the ALBI score associated with a 24% rise in mortality risk (8). Nonetheless, the ALBI score is confined to measurements of albumin and bilirubin alone and may not fully capture disease severity, particularly concerning inflammatory and coagulation factors integral to HF pathogenesis. Platelets play a critical role in HF by contributing to inflammation and microthrombi formation, exacerbating myocardial injury and cardiac dysfunction. Prior evidence has shown that platelet indices are closely related to HF severity, with low platelet counts and abnormal platelet-to-leukocyte ratios predicting worse outcomes (9). Moreover, composite coagulation scores incorporating platelet count have been associated with a markedly increased short-term mortality risk in critically ill HF patients (10). These findings suggest that platelet abnormalities not only reflect impaired hemostasis but also indicate ongoing inflammation and thrombosis in HF. Thus, the platelet-albumin-bilirubin (PALBI) score integrates platelet counts to potentially offer a more comprehensive prognostic assessment.

Originally developed for hepatocellular carcinoma to predict hepatic functional reserve and survival outcomes (11), recent research indicates PALBI's prognostic utility extends to other conditions. For instance, elevated PALBI scores have been strongly linked to higher 30-day mortality rates among individuals suffering from acute respiratory distress syndrome (12). However, the predictive value of the PALBI score in HF patients admitted to the ICU has not been clearly established.

This study, therefore, sought to assess the ability of the PALBI score to predict both short-term and long-term mortality in ICU-admitted HF patients, offering clinicians a more accurate prognostic tool.

## 2 Materials and methods

### 2.1 Data source

This retrospective analysis utilized data extracted from the MIMIC-IV v3.1 database, a widely recognized public ICU dataset managed by the Laboratory for Computational Physiology at MIT. It provides comprehensive, high-quality clinical data from patients admitted to the ICUs at Beth Israel Deaconess Medical Center (13). One of the study authors, Zhantao Cao, obtained authorized access to the data (Certification number: 14336451) and conducted data extraction in compliance with established data usage guidelines.

### 2.2 Participants

**Figure 1:**
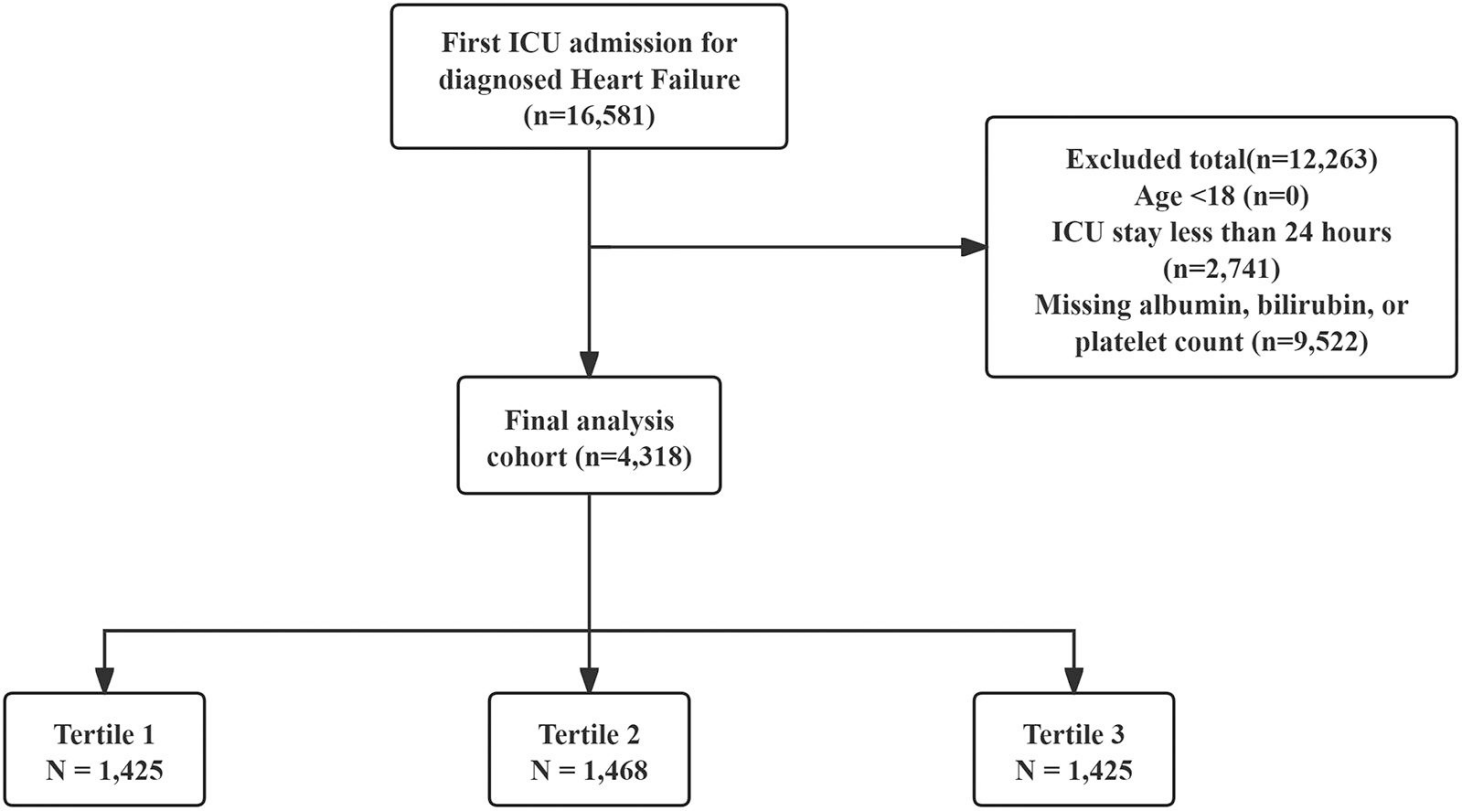
Patient screening flow from the MIMIC database.

Patients included were those with a first ICU admission and a HF diagnosis identified using ICD-9 and ICD-10 codes. Exclusion was based on the following conditions: (1) patients aged less than 18 years at initial admission; (2) patients hospitalized in the ICU for under 24 h; (3) patients without key laboratory results (albumin, bilirubin, or platelet counts). In total, 4,318 patients were included in the study and categorized into three groups based on PALBI score tertiles. The patient selection process is illustrated in Figure 1.

### 2.3 Data collection

Data were extracted using Structured Query Language (SQL) via pgAdmin (version 4). The following variables were collected within the first 24 h of ICU admission: (1) demographic information: age, gender, and ethnicity; (2) vital signs: heart rate, respiratory rate (RR), systolic blood pressure (SBP), diastolic blood pressure (DBP), body temperature, and peripheral oxygen saturation (SpO2); (3) comorbid conditions: myocardial infarction (MI), atrial fibrillation (AF), cerebrovascular disease (CeVD), chronic obstructive pulmonary disease (COPD), acute kidney injury (AKI), diabetes, and hypertension; (4) laboratory values: red blood cells (RBC), white blood cells (WBC), platelets, red blood cell distribution width (RDW), sodium, potassium, calcium, albumin, total bilirubin, blood urea nitrogen (BUN), creatinine, anion gap, international normalized ratio (INR), prothrombin time (PT), partial thromboplastin time (PTT), and urine output; (5) prescribed medications: angiotensin-converting enzyme inhibitors (ACEI), angiotensin II receptor blockers (ARB), beta-blockers, and statins; (6) medical interventions: mechanical ventilation (MV) and continuous renal replacement therapy (CRRT); and (7) assessment scores: Charlson Comorbidity Index (CCI), Oxford Acute Severity of Illness Score (OASIS), Sequential Organ Failure Assessment (SOFA), and Left Ventricular Ejection Fraction (LVEF). The PALBI score was calculated as: PALBI score = 2.02 × log_10_ bilirubin − 0.37 × (log_10_ bilirubin)^2^ − 0.04 × albumin − 3.48 × log_10_ platelets + 1.01 × (log_10_ platelets)^2^ (14), with bilirubin (μmol/L), albumin (g/L), and platelet counts (k/μl) (12).

To reduce possible bias, variables with over 20% missing values were excluded, whereas those missing less than 20% were imputed using the “mice” package in R (Supplementary Table S1). Considering the clinical importance of LVEF, we adopted the missing-indicator method despite the relatively high proportion of missing values: LVEF was entered into the regression model as a four-category variable (≥50%, 40%–49%, <40%, missing).

### 2.4 Clinical outcomes

The main outcome assessed was all-cause mortality within 30 days after ICU admission, while the secondary endpoint was overall mortality at 360 days post-admission.

### 2.5 Statistical analysis

Continuous variables were presented as the mean ± standard deviation (mean ± SD) when normally distributed, and as the median with interquartile range (IQR) for non-normally distributed data. Categorical variables were expressed as frequencies and percentages. The normality of continuous variables was tested using the Kolmogorov–Smirnov test. For normally distributed data, group comparisons were performed using independent *t*-tests or one-way ANOVA. For non-normally distributed variables, the Mann–Whitney *U* test or Kruskal–Wallis test was used as appropriate.

Kaplan–Meier survival curves were constructed to evaluate cumulative event rates across different PALBI score groups, with differences between groups assessed using the log-rank test. Additionally, univariate Cox regression analysis based on the proportional hazards model was performed to identify potential predictors of all-cause mortality.

Cox proportional hazards models were used to calculate hazard ratios (HRs) and 95% confidence intervals (CIs) to assess the relationship between PALBI scores and overall mortality, with adjustments for potential confounding factors. Variables with *p* < 0.05 in univariate analysis were included in the multivariate analysis. Clinically relevant variables with significant prognostic implications were also included in multivariate models as follows: Model 1 included no covariate adjustments; Model 2 accounted for age, gender, and race; Model 3 incorporated additional adjustments for age, gender, race, SBP, MI, AF, CeVD, AKI, WBC count, serum potassium, creatinine, INR, beta-blocker use, and LVEF categories.

To assess potential multicollinearity among covariates, we calculated the generalized variance inflation factor (GVIF) for all variables included in the multivariable models. A GVIF^(1/2Df) < 2 was considered acceptable, indicating no concerning collinearity. Detailed results are provided in Supplementary Table S2.

In addition, restricted cubic spline (RCS) regression with three knots was applied to investigate possible nonlinear associations between baseline PALBI scores and the risk of all-cause mortality at both 30 and 360 days. PALBI scores were analyzed as continuous and ordinal categorical variables, the lowest tertile was designated as the reference category in comparisons.To assess the reliability of the PALBI score in forecasting overall mortality, stratified analyses were conducted by gender, ethnicity, CeVD, COPD, MV and LVEF categories. Interaction effects between the PALBI score and the stratification variables were evaluated using likelihood ratio tests. A two-sided *p*-value of less than 0.05 was regarded as statistically significant. All statistical analyses were conducted using R software (version 4.4.2).

### 2.6 Construction and assessment of the prognostic models

The data were randomly split into training and validation sets in a 7:3 ratio. To manage a large number of features, the least absolute shrinkage and selection operator (LASSO) was used for feature selection in the training set. A five-fold cross-validation method was employed to determine the optimal regularization parameter. By shrinking coefficients, LASSO selects features with greater predictive contributions and eliminates redundant variables, thereby effectively achieving feature selection and dimensionality reduction.

The chosen variables were then fed into multiple machine learning models to estimate the 30-day all-cause mortality risk among HF patients. Specifically, each selected feature set was applied to multiple models including decision tree (DT), ridge classifier (Ridge, a linear model with L2 regularization), K-nearest neighbors (KNN), light gradient boosting machine (LightGBM), random forest (RF), extreme gradient boosting (XGBoost), support vector machine (SVM), and multilayer perceptron (MLP). Hyperparameter tuning was conducted to optimize model performance. Model training was conducted on the training dataset, while the validation dataset was employed to evaluate predictive accuracy. The discriminative ability of each model was evaluated using the area under the receiver operating characteristic curve (AUC-ROC). To assess the clinical applicability of the models, decision curve analysis (DCA) was conducted, and calibration plots were used to compare the predicted probabilities with the actual outcomes.

## 3 Results

A total of 4,318 HF patients admitted to the ICU were included in the study. The median age was 73.25 years [interquartile range (IQR): 62.98–82.38], and 57% were male. The overall median PALBI score was −6.05 (IQR: −6.60 to −5.41). The all-cause mortality rates at 30 and 360 days were observed to be 24% and 44%, respectively (Table 1).

**Table 1:** Characteristics and outcomes of participants categorized by PALBI tertiles.

Characteristic	Overall *N* = 4,318	T1 *N* = 1,425	T2 *N* = 1,468	T3 *N* = 1,425	*p*-value
Age, Median (Q1, Q3)	**73.25 (62.98, 82.38)**	**72.33 (62.49, 81.26)**	**74.36 (63.86, 83.36)**	**73.08 (62.30, 82.20)**	**<0.001**
Gender, *n* (%)					**<0.001**
Female	**1,860 (43%)**	**755 (53%)**	**614 (42%)**	**491 (34%)**	
Male	**2,458 (57%)**	**670 (47%)**	**854 (58%)**	**934 (66%)**	
Race, *n* (%)					0.095
Other	1,426 (33%)	440 (31%)	507 (35%)	479 (34%)	
White	2,892 (67%)	985 (69%)	961 (65%)	946 (66%)	
Heart rate (bpm)	**89.00 (76.00, 104.00)**	**90.00 (76.00, 105.00)**	**88.00 (74.00, 102.00)**	**89.00 (77.00, 105.00)**	**0.018**
SBP (mmHg)	**120.00 (103.00, 137.00)**	**123.00 (107.00, 141.00)**	**121.00 (104.00, 139.00)**	**114.00 (100.00, 132.00)**	**<0.001**
DBP (mmHg)	**67.00 (56.00, 79.00)**	**68.00 (56.00, 81.00)**	**68.00 (56.00, 79.50)**	**66.00 (55.00, 78.00)**	**0.003**
RR (bpm)	20.00 (17.00, 25.00)	20.00 (17.00, 25.00)	20.00 (17.00, 24.00)	20.00 (16.00, 25.00)	0.339
Temperature (℃)	36.67 (36.39, 37.06)	36.72 (36.39, 37.06)	36.67 (36.44, 37.06)	36.67 (36.39, 37.00)	0.152
SpO2 (%)	**97.00 (94.00, 99.00)**	**97.00 (94.00, 99.00)**	**97.00 (94.00, 99.00)**	**97.00 (94.00, 100.00)**	**0.044**
SOFA	**1.00 (0.00, 4.00)**	**1.00 (0.00, 3.00)**	**1.00 (0.00, 3.00)**	**2.00 (1.00, 5.00)**	**<0.001**
OASIS	**34.00 (28.00, 40.00)**	**33.00 (28.00, 40.00)**	**33.00 (28.00, 39.00)**	**34.00 (28.00, 42.00)**	**<0.001**
CCI	**7.00 (5.00, 9.00)**	**7.00 (5.00, 9.00)**	**7.00 (5.00, 9.00)**	**7.00 (5.00, 9.00)**	**0.012**
MI, *n* (%)	**1,522 (35%)**	**544 (38%)**	**525 (36%)**	**453 (32%)**	**0.002**
AF, *n* (%)	**2,181 (51%)**	**624 (44%)**	**789 (54%)**	**768 (54%)**	**<0.001**
CeVD, *n* (%)	**631 (15%)**	**221 (16%)**	**230 (16%)**	**180 (13%)**	**0.035**
COPD, *n* (%)	**1,454 (34%)**	**544 (38%)**	**477 (32%)**	**433 (30%)**	**<0.001**
AKI, *n* (%)	**3,697 (86%)**	**1,185 (83%)**	**1,276 (87%)**	**1,236 (87%)**	**0.005**
Diabetes, *n* (%)	**1,767 (41%)**	**658 (46%)**	**590 (40%)**	**519 (36%)**	**<0.001**
Hypertension, *n* (%)	**3,315 (77%)**	**1,126 (79%)**	**1,160 (79%)**	**1,029 (72%)**	**<0.001**
RBC (10^9^/L)	**3.55 (3.01, 4.16)**	**3.55 (3.08, 4.11)**	**3.68 (3.08, 4.28)**	**3.43 (2.79, 4.07)**	**<0.001**
WBC (10^9^/L)	**11.10 (7.90, 16.00)**	**12.50 (9.10, 17.10)**	**10.80 (7.80, 15.20)**	**10.10 (7.00, 15.30)**	**<0.001**
Platelet (10^9^/L)	**197.00 (142.00, 267.00)**	**284.00 (229.00, 347.00)**	**193.00 (160.00, 237.00)**	**127.00 (88.00, 165.00)**	**<0.001**
RDW	**15.20 (14.00, 17.00)**	**14.90 (13.90, 16.70)**	**15.00 (13.90, 16.50)**	**15.80 (14.50, 17.90)**	**<0.001**
Sodium (mmol/L)	138.00 (135.00, 141.00)	138.00 (135.00, 141.00)	138.00 (135.00, 141.00)	138.00 (134.00, 141.00)	0.202
Potassium (mmol/L)	**4.30 (3.80, 4.80)**	**4.30 (3.90, 4.90)**	**4.30 (3.80, 4.70)**	**4.20 (3.80, 4.80)**	**0.024**
Calcium (mmol/L)	**8.40 (7.90, 8.90)**	**8.50 (8.00, 9.00)**	**8.50 (8.00, 9.00)**	**8.30 (7.70, 8.80)**	**<0.001**
Albumin (g/L)	**3.20 (2.80, 3.60)**	**3.20 (2.80, 3.60)**	**3.30 (2.90, 3.60)**	**3.10 (2.70, 3.50)**	**<0.001**
Bilirubin total (μmol/L)	**0.70 (0.40, 1.23)**	**0.40 (0.30, 0.50)**	**0.70 (0.50, 0.94)**	**1.60 (1.00, 2.80)**	**<0.001**
PALBI	**−6.05 (−6.60, −5.41)**	**−6.81 (−7.12, −6.60)**	**−6.05 (−6.22, −5.87)**	**−5.03 (−5.40, −4.44)**	**<0.001**
BUN (mg/dl)	**30.00 (19.00, 48.00)**	**28.00 (19.00, 46.00)**	**29.00 (18.00, 46.00)**	**33.00 (21.00, 52.00)**	**<0.001**
Creatinine (mg/dl)	**1.40 (0.90, 2.20)**	**1.30 (0.90, 2.20)**	**1.30 (0.90, 2.20)**	**1.50 (1.00, 2.30)**	**<0.001**
Anion gap (mmol/L)	**15.00 (13.00, 18.00)**	**15.00 (13.00, 18.00)**	**15.00 (13.00, 18.00)**	**15.00 (13.00, 19.00)**	**0.008**
INR	**1.30 (1.20, 1.70)**	**1.20 (1.10, 1.50)**	**1.30 (1.20, 1.60)**	**1.60 (1.30, 2.10)**	**<0.001**
PT (S)	**14.80 (12.90, 19.00)**	**13.80 (12.30, 16.50)**	**14.30 (12.70, 17.75)**	**17.00 (14.30, 22.90)**	**<0.001**
PTT (S)	**33.10 (28.40, 43.30)**	**31.80 (27.80, 41.30)**	**32.60 (28.05, 43.30)**	**34.90 (29.90, 45.70)**	**<0.001**
Urine Output (ml)	**1,375.00 (747.00, 2,385.00)**	**1,500.00 (850.00, 2,460.00)**	**1,416.50 (796.50, 2,445.00)**	**1,230.00 (600.00, 2,152.00)**	**<0.001**
LVEF, *n* (%)					**0.010**
≥50%	**1,016** (**23.53)**	**347** (**24.35)**	**349** (**23.77)**	**320** (**22.46)**	
40%–49%	**328** (**7.60)**	**113** (**7.93)**	**117** (**7.97)**	**98** (**6.88)**	
<40%	**647** (**14.98)**	**172** (**12.07)**	**231** (**15.74)**	**244** (**17.12)**	
Missing	**2,327** (**53.89)**	**793** (**55.65)**	**771** (**52.52)**	**763** (**53.54)**	
ACEI/ARB *n* (%)	**1,451 (34%)**	**543** (**38%)**	**529** (**36%)**	**379** (**27%)**	**<0**.**001**
Beta-blockers *n* (%)	**2,948 (68%)**	**1,015 (71%)**	**1,055 (72%)**	**878** (**62%)**	**<0**.**001**
Statin *n* (%)	**2,095 (49%)**	**762** (**53%)**	**753** (**51%)**	**580** (**41%)**	**<0**.**001**
MV *n* (%)	3,706 (86%)	1,230 (86%)	1,259 (86%)	1,217 (85%)	0.781
CRRT, *n* (%)	**427** (**10%)**	**103** (**7%)**	**128** (**9%)**	**196** (**14%)**	**<0**.**001**
Hospital stay (day)	**9.99** (**5.99, 16.97)**	**9.80** (**5.88, 16.74)**	**9.74** (**6.01, 16.45)**	**10.72** (**6.08, 18.65)**	**0**.**036**
Hospital mortality *n* (%)	**878** (**20%)**	**231** (**16%)**	**274** (**19%)**	**373** (**26%)**	**<0**.**001**
ICU stay (day)	3.35 (1.95, 6.31)	3.17 (1.91, 6.01)	3.40 (1.94, 6.27)	3.53 (1.98, 6.86)	0.108
ICU mortality *n* (%)	**607** (**14%)**	**154** (**11%)**	**191** (**13%)**	**262** (**18%)**	**<0**.**001**
30-day all-cause Mortality *n* (%)	**1,048 (24%)**	**285** (**20%)**	**334** (**23%)**	**429** (**30%)**	**<0**.**001**
360-day all-cause Mortality *n* (%)	**1,902 (44%)**	**557** (**39%)**	**611** (**42%)**	**734** (**52%)**	**<0**.**001**

T1 (−8.48 to −6.41), T2 (−6.41 to −5.65), T3 (−5.65 to −0.62).

Bold values indicate statistically significant results.

SBP, systolic blood pressure; DBP, diastolic blood pressure; RR, respiratory rate; SpO2, peripheral capillary oxygen saturation; SOFA, Sequential Organ Failure Assessment; OASIS, Oxford Acute Severity of Illness Score; CCI, Charlson Comorbidity Index; MI, myocardial infarction; AF, atrial fibrillation; CeVD, cerebrovascular disease; COPD, chronic obstructive pulmonary disease; AKI, acute kidney injury; RBC, red blood cell count; WBC, white blood cell count; RDW, red cell distribution width; BUN, blood urea nitrogen; INR, international normalized ratio; PT, prothrombin time; PTT, partial thromboplastin time; LVEF, Left Ventricular Ejection Fraction; ACEI/ARB, angiotensin-converting enzyme inhibitor/angiotensin receptor blocker; MV, mechanical ventilation; CRRT, continuous renal replacement therapy; PALBI, platelet–albumin–bilirubin score.

### 3.1 Baseline characteristics

Table 1 presents the baseline characteristics of ICU-admitted HF patients, stratified by PALBI tertiles. Based on PALBI scores at ICU admission, patients were categorized into three groups: T1 (–8.48 to −6.41), T2 (–6.41 to −5.65), and T3 (–5.65 to −0.62). The median PALBI scores for the T1, T2, and T3 groups were −6.81 (IQR: −7.12 to −6.60), −6.05 (IQR: −6.22 to −5.87), and −5.03 (IQR: −5.40 to −4.44), respectively. Compared to individuals in the lowest PALBI tertile, those in the highest tertile were generally older and more frequently male, had lower systolic and diastolic blood pressures, reduced oxygen saturation, and elevated SOFA, OASIS, and CCI scores. In terms of cardiac function, there was a statistically significant difference in the distribution of LVEF categories among the groups (*p* = 0.010), with patients in the highest PALBI tertile showing a higher proportion of LVEF <40% compared to those in the lowest tertile. The prevalence of AF and AKI was notably higher in the high PALBI group, whereas the prevalence of MI, CeVD, COPD, diabetes, and hypertension was lower. In terms of laboratory findings, individuals in the high PALBI group showed reduced platelet counts, lower serum albumin and calcium levels, alongside elevated RDW, WBC, total bilirubin, BUN, creatinine, INR, PT, and PTT. With respect to treatment modalities, a higher proportion of patients in this group underwent CRRT, whereas the use of ACEI/ARB, beta-blockers, and statins was markedly lower. Clinically, those in the highest PALBI tertile experienced significantly greater all-cause mortality at both 30 days (20% vs. 23% vs. 30%, *p* < 0.001) and 360 days (39% vs. 42% vs. 52%, *p* < 0.001) compared with patients in the lower tertiles.

### 3.2 Clinical outcomes

**Figure 2:**
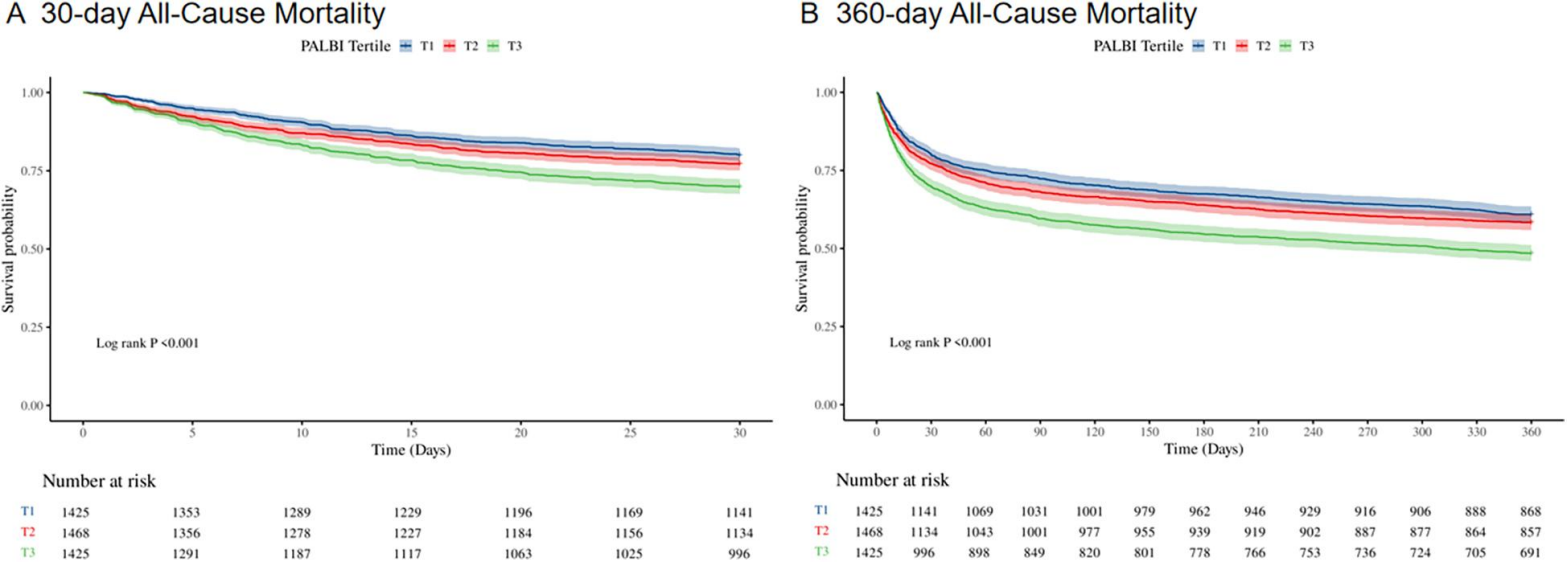
Kaplan–Meier survival curves for 30-day **(A)** and 360-day **(B)** all-cause mortality across PALBI score tertiles in ICU heart failure patients.

Figure 2 presents Kaplan–Meier survival curves depicting primary outcomes across different PALBI tertiles. Patients in the highest PALBI tertile exhibited significantly reduced survival probabilities at both 30 days (Figure 2A) and 360 days (Figure 2B) compared to those in the lowest tertile (log-rank test, *p* < 0.001).

Supplementary Table S3 summarizes the univariate Cox regression findings for all-cause mortality among ICU-admitted HF patients. Variables with *P*-values below 0.05 in the univariate analysis, together with clinically significant predictors identified by physicians, were incorporated into the multivariate Cox regression model for further assessment. The final multivariate model revealed several independent factors that were significantly linked to all-cause mortality, including age, gender, race, SBP, MI, AF, CeVD, AKI, WBC count, serum potassium, creatinine, INR, beta-blocker use, and LVEF categories.

To examine the relationship between PALBI score and all-cause mortality, multivariable Cox regression analysis was performed, as shown in Table 2. When analyzed as a continuous variable, the PALBI score was significantly associated with 30-day mortality risk across all models: in the unadjusted model [HR = 1.24; 95% CI: 1.18–1.31; *p* < 0.001], the partially adjusted model [HR = 1.28; 95% CI: 1.21–1.36; *p* < 0.001], and the fully adjusted model [HR = 1.30; 95% CI: 1.17–1.44; *p* < 0.001]. A comparable pattern was seen for 360-day mortality. When the PALBI score was analyzed as a categorical variable based on tertiles, patients in the highest tertile (T3) faced a substantially greater risk of 30-day all-cause death than those in the lowest tertile (T1), across all models: Model 1 [HR = 1.62; 95% CI: 1.40–1.88; *p* < 0.001], Model 2 [HR = 1.58; 95% CI: 1.36–1.84; *p* < 0.001], and Model 3 [HR = 1.36; 95% CI: 1.12–1.64; *p* = 0.002]. A similar gradient was noted for 360-day mortality, with the PALBI score displaying a progressive increase in death risk: Model 1 [HR = 1.49; 95% CI: 1.34–1.67; *p* < 0.001], Model 2 [HR = 1.47; 95% CI: 1.31–1.64; *p* < 0.001], and Model 3 [HR = 1.22; 95% CI: 1.03–1.44; *p* = 0.019]. These results support a dose–response association between the PALBI score and all-cause mortality among ICU HF patients.

**Table 2:** Association between PALBI score and 30-day and 360-day all-cause mortality. .

Variables	Model 1		Model 2		Model 3	
	HR (95% CI)	*p*	HR (95% CI)	*p*	HR (95% CI)	*p*
30-day Mortality						
PALBI	1.24 (1.18–1.31)	**<** **.** **001**	1.28 (1.21–1.36)	**<** **.** **001**	1.30 (1.17–1.44)	**<** **.** **001**
PALBI Tertile						
T1	1.00 (Reference)		1.00 (Reference)		1.00 (Reference)	
T2	1.17 (1.01–1.37)	**0** **.** **049**	1.10 (0.94–1.29)	**0** **.** **228**	0.99 (0.82–1.19)	**0** **.** **902**
T3	1.62 (1.40–1.88)	**<** **.** **001**	1.58 (1.36–1.84)	**<** **.** **001**	1.36 (1.12–1.64)	**0** **.** **002**
360-day Mortality						
PALBI	1.22 (1.17–1.27)	**<** **.** **001**	1.25 (1.20–1.31)	**<** **.** **001**	1.19 (1.09–1.31)	**<** **.** **001**
PALBI Tertile						
T1	1.00 (Reference)		1.00 (Reference)		1.00 (Reference)	
T2	1.11 (0.99–1.24)	**0** **.** **080**	1.06 (0.94–1.19)	**0** **.** **344**	0.98 (0.83–1.15)	**0** **.** **781**
T3	1.49 (1.34–1.67)	**<** **.** **001**	1.47 (1.31–1.64)	**<** **.** **001**	1.22 (1.03–1.44)	**0** **.** **019**

Model 1: Crude.

Model 2: Adjust for Age, Gender, Race.

Model 3: Adjust for Age, Gender, Race, SBP, Myocardial infarct, AF, Cerebrovascular disease, AKI, WBC, Potassium, Creatinine, INR, Beta blockers, LVEF category.

Bold values indicate statistically significant results.

PALBI, platelet–albumin–bilirubin score; HR, hazard ratio; CI, confidence interval; SBP, systolic blood pressure; AF, atrial fibrillation; AKI, acute kidney injury; WBC, white blood cell count; INR, international normalized ratio; LVEF, Left Ventricular Ejection Fraction.

**Figure 3:**
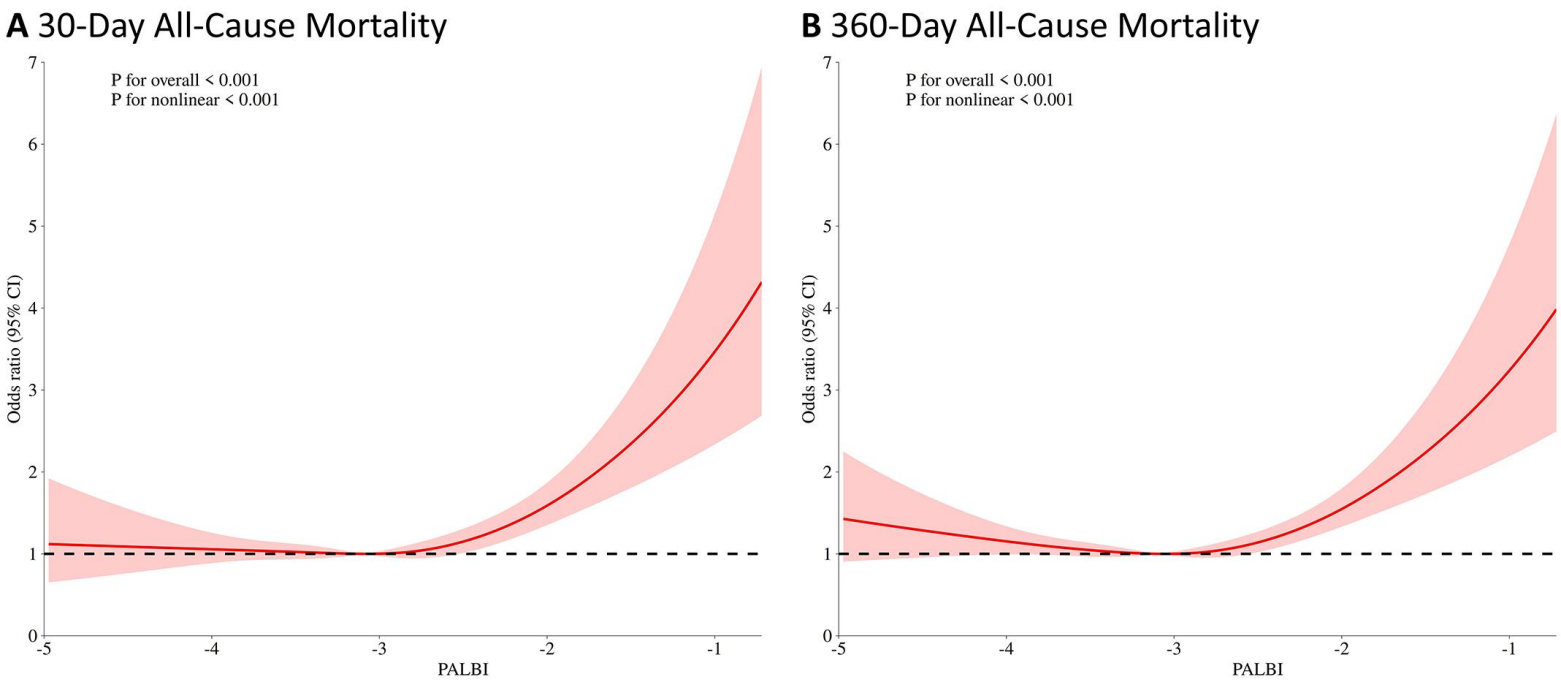
Restricted cubic spline (RCS) analysis of the association between PALBI score and 30-day **(A)** and 360-day **(B)** all-cause mortality.

In addition, RCS regression analysis demonstrated a nonlinear relationship between the PALBI score and all-cause mortality at both 30 and 360 days. As the PALBI score increased, the risk of death escalated significantly. This nonlinear association was statistically significant for both 30-day mortality (*p* for nonlinear <0.001) and 360-day mortality (*p* for nonlinear <0.001), as shown in Figure 3.

### 3.3 Subgroup analysis

**Figure 4:**
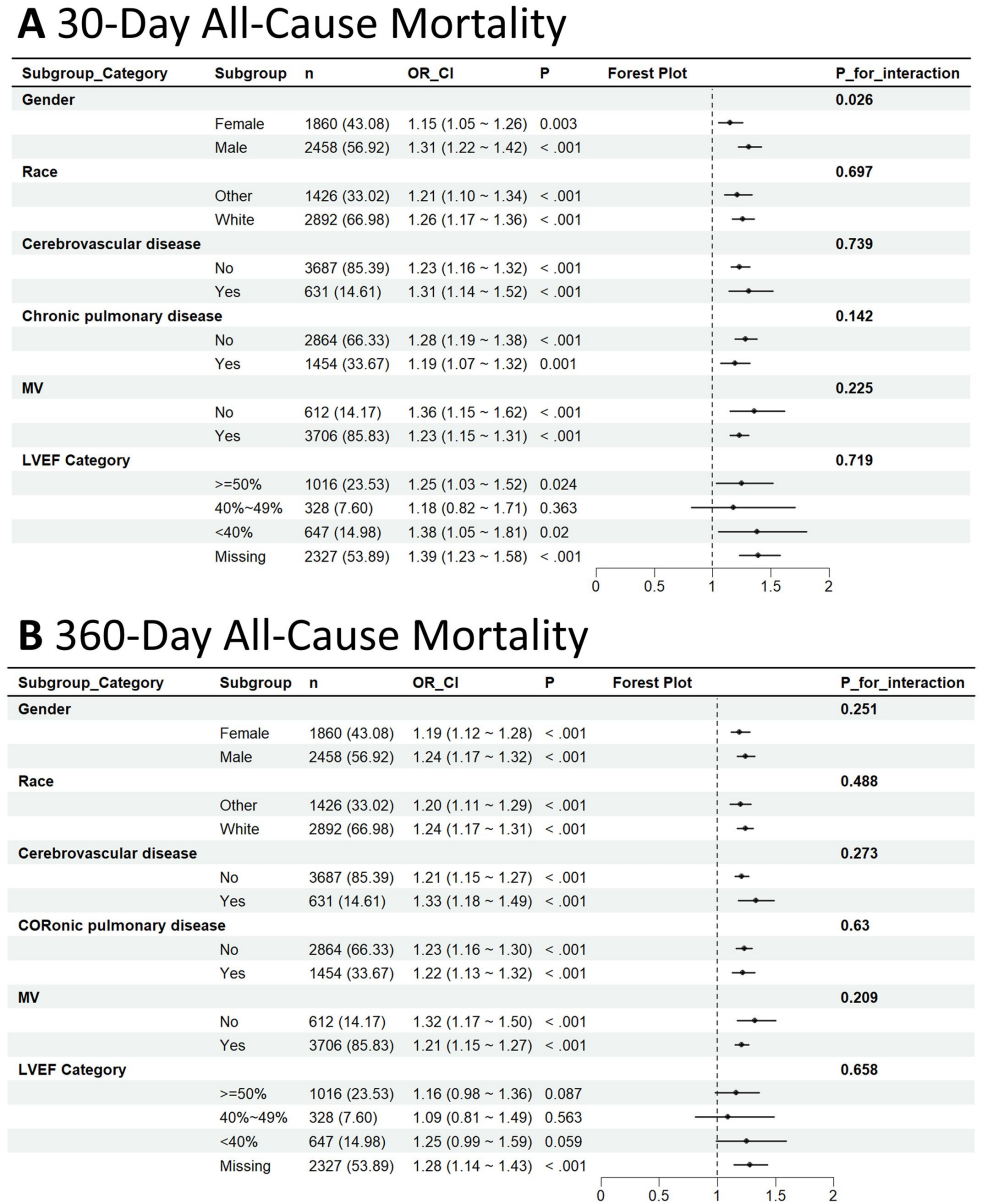
Subgroup analysis of the association between PALBI score and 30-day **(A)** and 360-day all-cause mortality **(B)**.

Subgroup analyses reinforced the stability of the link between higher PALBI scores and elevated mortality risk across various clinical subgroups, as depicted in Figure 4. In the analysis of mortality at 30 and 360 days, the association remained significant across strata defined by gender, race, presence of CeVD, COPD, and MV use. When stratified by LVEF, patients with reduced LVEF (<40%) showed a more pronounced association between PALBI and mortality compared with those with preserved LVEF, although no statistically significant interaction was observed.

Among patients who died within 30 days, the association was more pronounced in males [HR = 1.31; 95% CI: 1.22–1.42] than in females [HR = 1.15; 95% CI: 1.05–1.26], with a statistically notable interaction by gender (interaction *p*-value = 0.026). Similar consistent trends were observed within the CeVD, COPD, and MV subgroups; however, no statistically significant interactions were identified, as all interaction *p*-values exceeded 0.05.

For 360-day mortality, the relationship between PALBI score and death risk remained consistent across all examined subgroups, with no indication of significant interaction effects. These results suggest that the PALBI score serves as a reliable and robust predictor of all-cause mortality among ICU-admitted HF patients, irrespective of their clinical subgroup features.

### 3.4 Feature selection

**Figure 5:**
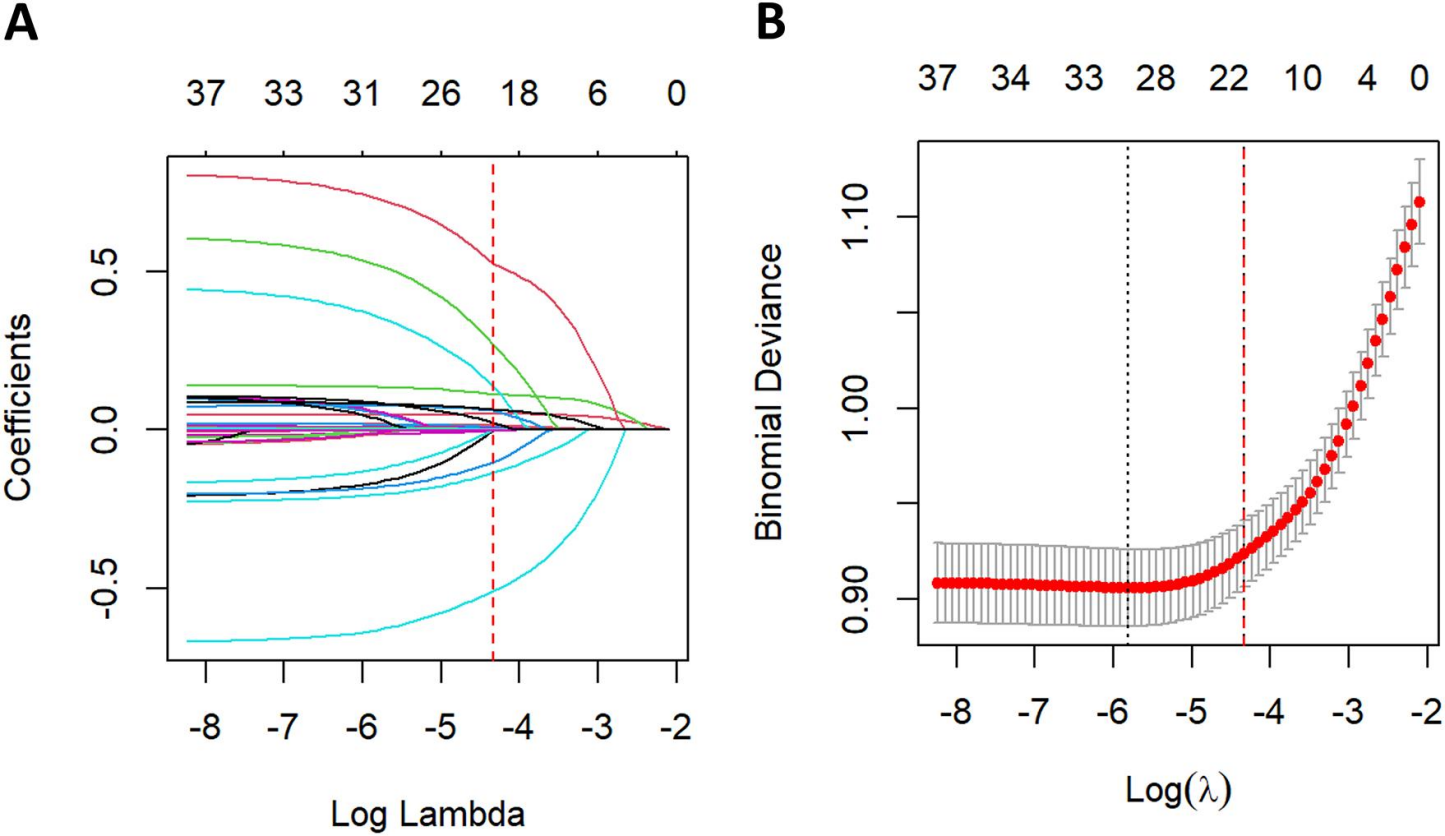
Lasso regression-based variable screening.

Feature selection using LASSO regression was performed in the training cohort, as illustrated in Figure 5. Five-fold cross-validation was employed to identify the optimal penalization parameter (lambda) during model construction. Ultimately, 20 variables were selected as most predictive of all-cause mortality: age, SBP, RR, temperature, OASIS score, CCI, CeVD, AKI, ACEI/ARB use, beta-blocker use, CRRT, WBC, RDW, PALBI score, BUN, PTT, urine output, SpO2, PT, and LVEF category.

### 3.5 Construction and validation of the risk prediction model

**Figure 6:**
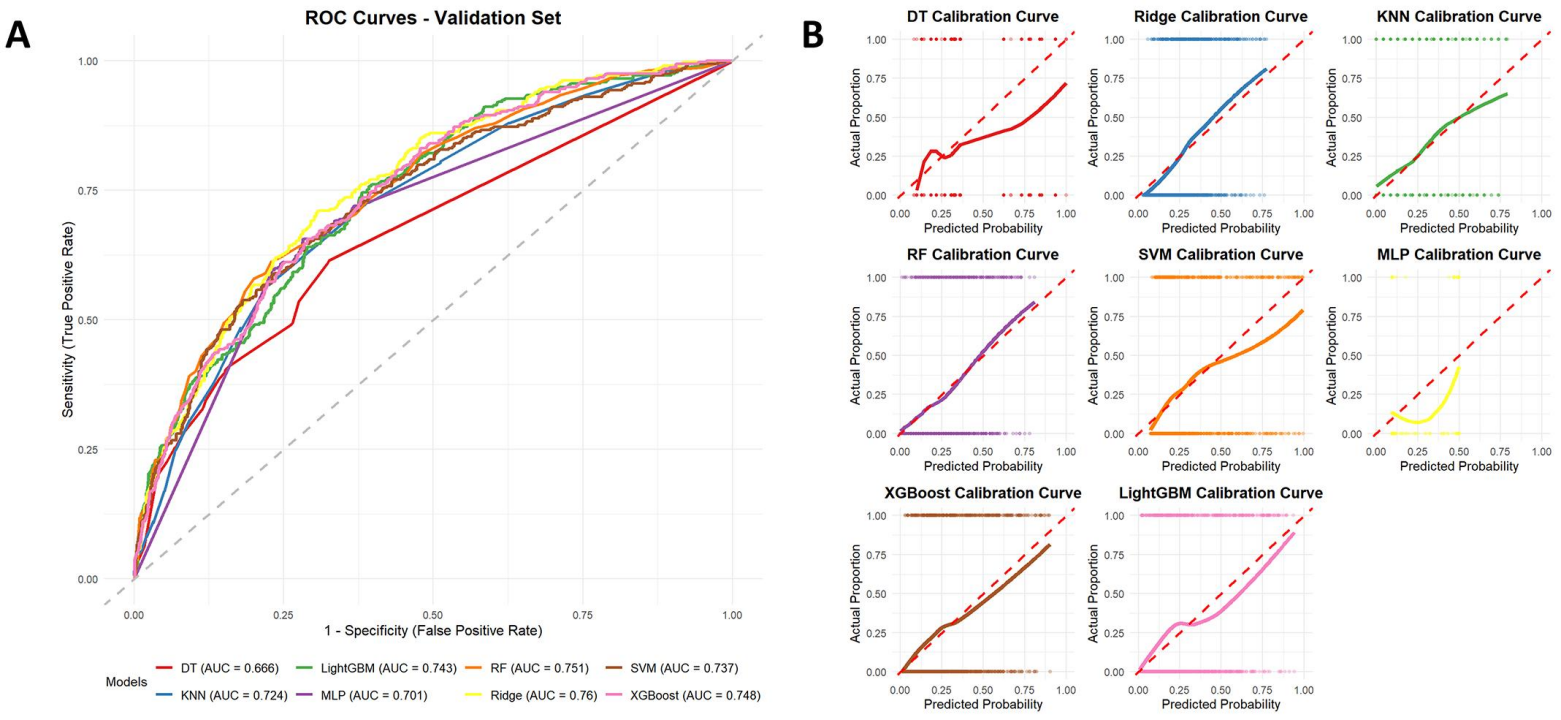
Receiver operating characteristic (ROC) curves of different machine learning models for predicting all-cause mortality on the test dataset **(A)**, with ridge regression showing the best performance (AUC = 0.76). Calibration curves of different machine learning models for predicting all-cause mortality **(B)**.

Figure 6A displays the ROC curves corresponding to each machine learning algorithm, with performance assessed by the AUC. The AUC values were as follows: Ridge (0.760), RF (0.751), XGBoost (0.748), LightGBM (0.743), SVM (0.737), KNN (0.724), MLP (0.701), and DT (0.666). Figure 6B illustrates the calibration plots for each model evaluated on the test dataset. Calibration curves indicated that ensemble models (RF, XGBoost, and LightGBM) and Ridge provided closer agreement between predicted and observed probabilities. Based on the decision curve analysis shown in Supplementary Figure S1, most models, particularly Ridge, RF, XGBoost, and LightGBM, exhibited evident net clinical benefit across a wide range of threshold probabilities, suggesting strong potential for clinical applicability.

## 4 Discussion

This investigation examined the association between PALBI scores and all-cause mortality in ICU-admitted patients with HF. The results indicated that higher PALBI scores were strongly associated with increased 30-day and 360-day all-cause mortality. In our study, patients in the highest PALBI tertile had approximately 1.36 times higher risk of 30-day mortality and 1.22 times higher risk of 360-day mortality compared with those in the lowest tertile. RCS analysis confirmed a nonlinear relationship between PALBI and mortality risk at both 30 and 360 days, with risk rising steeply at higher PALBI scores. The machine-learning suite identified patients at risk of 30-day mortality with favorable accuracy, led by Ridge regression (AUC = 0.76). These findings suggest that incorporating PALBI into risk stratification may improve prognostic assessment in critically ill HF patients.

The PALBI score, which integrates platelet count, serum albumin, and total bilirubin, was initially designed to evaluate hepatic function and forecast outcomes in individuals with hepatocellular carcinoma (15). While its initial application was in hepatic diseases, the use of PALBI in the context of HF represents a promising approach. Prior studies have validated the ALBI score's prognostic utility in HF across diverse populations, including acute HF (16), elderly with decompensated HF (17), ICU admissions (8), reduced ejection fraction (18), CRT recipients (19), and in-hospital mortality prediction alongside N-terminal pro–B-type natriuretic peptide (NT-pro BNP) (20), underscoring hepatic dysfunction's prognostic relevance. However, emerging evidence suggests that PALBI may offer prognostic advantages in HF beyond those of ALBI. In HF, platelet indices have been repeatedly linked to disease severity and outcomes, and composite coagulation scores that include platelet count strongly stratify mortality. For example, Noeva et al. showed that worse cardiac function and higher HF event rates were associated with low platelet count and high platelet-to-leukocyte ratios (9). Similarly, Tang et al. found that a higher “coagulation disorder” score (based on platelet count, INR, and APTT) predicted nearly double the 30-day mortality in critically ill CHF patients (10). By contrast, ALBI (bilirubin + albumin) omits this dimension. Empirically, PALBI has shown broader predictive accuracy than ALBI in analogous settings. In transcatheter aortic valve replacement patients (a population with cardiohepatic interactions), Duan et al. found that adding the PALBI score to the standard STS risk model significantly improved discrimination of mortality (21). They noted that most cardiac risk scores do not explicitly account for liver function or coagulopathy—gaps that PALBI helps to fill. However, studies specifically evaluating the utility of the PALBI score in HF remain limited. Our findings suggest that PALBI holds independent prognostic significance in ICU-admitted patients with HF, thus extending its potential application beyond liver-related conditions.

The relationship of elevated PALBI values with mortality among individuals with HF may be explained by multiple underlying pathophysiological mechanisms. Fundamentally, the PALBI score is a composite marker that reflects hepatic dysfunction—through albumin and bilirubin—and hematologic status via platelet count. HF often gives rise to what is known as cardiohepatic syndrome or congestive hepatopathy, a condition marked by persistently elevated central venous pressure and diminished cardiac output, which in turn causes liver congestion, inadequate perfusion, and hepatic damage (4, 22). A direct consequence of this process is elevated serum bilirubin levels, which are indicative of cholestasis and have been associated with poor outcomes in HF, even with modest increases (23). Serum albumin levels are often decreased in HF patients due to factors such as hemodilution from fluid overload, malnutrition, and impaired hepatic synthesis. Earlier research has established hypoalbuminemia as a powerful indicator of adverse prognosis in HF. For instance, Chao and colleagues reported that lower albumin levels were significantly correlated with short-term mortality among ICU-admitted HF patients (24). In addition to bilirubin and albumin, the PALBI score incorporates platelet count. Thrombocytopenia in HF may result from several mechanisms, with a prominent one being portal hypertension and splenic sequestration due to cardiogenic cirrhosis, leading to reduced circulating platelet levels (25). Wang et al. reported that among ICU patients with acute HF, lower baseline platelet counts independently predicted in-hospital mo2rtality, and both markedly low and unusually high platelet levels were associated with unfavorable outcomes (26). In our study, elevated PALBI scores may result from various combinations of these abnormalities. For instance, thrombocytopenia combined with hypoalbuminemia and hyperbilirubinemia likely indicates severe HF with overt congestive hepatopathy. Alternatively, patients with moderately preserved platelet counts but profoundly low albumin and elevated bilirubin may also exhibit high PALBI scores. Both patterns reflect significant multisystem involvement. This may explain why PALBI, which includes platelet count, provides greater prognostic accuracy than ALBI in certain settings. Essentially, the PALBI score serves as a surrogate marker for the systemic burden of HF, capturing the extent of hepatic dysfunction (bilirubin), protein synthesis or nutritional reserve (albumin), and hematologic compromise (platelet count). Severe derangements in these parameters often indicate advanced disease with multiorgan involvement, which corresponds to elevated mortality risk. In summary, the link between PALBI and mortality in HF patients may stem from its ability to encapsulate key elements of hepatic injury, malnutrition, and potential coagulation abnormalities—all of which contribute synergistically to poor clinical outcomes.

In our subgroup analysis, we found a significant sex–PALBI interaction on 30-day mortality in HF: the hazard ratio was higher in men (HR = 1.31) than in women (HR = 1.15), suggesting increasing PALBI more strongly predicts mortality in men. This may be explained by that women with HF often have better outcomes and distinct clinical features (27). Men more commonly have ischemic HF with reduced ejection fraction, while women more often have HFpEF (28). Ischemic HF may exacerbate liver dysfunction via inflammation and oxidative stress, worsening outcomes in men. Hormonal differences (e.g., estrogen vs. testosterone) and immune responses also affect cardiac remodeling and prognosis (29). Men also have higher rates of alcohol misuse and metabolic syndrome (30, 31), which can further impair liver function. Clinically, an elevated PALBI in men may flag a high-risk subgroup requiring aggressive intervention, whereas PALBI is less predictive in women. Biologically, the known protective effects of estrogen may explain better outcomes in women, while the stronger systemic inflammatory response in men can exacerbate liver injury (32). These findings suggest a clinical need for different PALBI risk thresholds for male and female patients, guiding more personalized care, such as emphasizing anti-inflammatory management in men.Therefore, further multicenter trials are needed to validate these sex-based differences and mechanisms.

Our study carries important clinical implications by identifying the PALBI score as a robust and easily calculable prognostic tool for ICU HF patients. HF has long been acknowledged as a condition affecting multiple organ systems, and our findings reinforce the necessity of incorporating assessments of liver function and nutritional status into comprehensive risk stratification. One of the major advantages of the PALBI score is its simplicity—it is derived from routine laboratory tests, including platelet count, albumin, and bilirubin, all of which are commonly measured in hospitalized patients. This enables immediate application in clinical settings without additional cost or testing burden. To facilitate its clinical implementation for risk stratification in the ICU, we propose a specific cut-off value. Based on ROC curve analysis for 30-day mortality, a PALBI score threshold of −2.45 was identified (Youden's index). This threshold falls within the third tertile of our cohort. Using this ROC-derived value, patients can be dichotomized into high-risk (PALBI > −2.45) and low-risk (PALBI ≤ −2.45) groups. The use of this single, readily calculable threshold can provide clinicians with an immediate and objective means to identify high-risk HF patients upon ICU admission, potentially triggering closer monitoring or more intensive therapy. For ICU-admitted HF patients, a high PALBI score upon admission may alert clinicians to an elevated risk of mortality, warranting closer hemodynamic monitoring, more aggressive diuresis, or early multidisciplinary interventions, such as consultation with hepatology or nutritional support services. Such approaches may offer considerable benefit, since promptly recognizing high-risk individuals could enable the implementation of focused interventions—like nutritional support or anti-inflammatory treatments—that have the potential to enhance clinical outcomes. Another advantage of the PALBI score is its objectivity. Unlike other clinical scoring systems that incorporate subjective assessments—such as the Child-Pugh score or the NYHA classification for HF—the PALBI score is entirely laboratory-based, thereby minimizing interobserver variability and enhancing reproducibility.

Our study is the first to assess PALBI in ICU HF patients, extending its use beyond liver disease. Incorporating platelet count, PALBI remains a strong prognostic marker and may enhance risk stratification when combined with tools like APACHE II or BNP. Despite these strengths, our study has several limitations. First, the retrospective design based on a single-center database may introduce selection bias and limits causal inference. Second, while we accounted for numerous confounding factors in our multivariable models, residual confounding cannot be entirely ruled out. Third, due to the intrinsic limitations of the database, we were unable to include BNP or NT-pro BNP levels in our primary analysis, which may have affected the accuracy of both the association estimates and model predictions. Fourth, the database did not provide information on rehospitalization or causes of death (cardiovascular vs. non-cardiovascular), so our endpoints were restricted to all-cause mortality. This limited the scope of clinical interpretation. Another limitation is that our study was based on a single-center database from the United States, which may reduce the generalizability of the findings to other ethnic groups and healthcare systems. Therefore, future research should aim to validate these results in multicenter studies or prospective cohorts to confirm their broader applicability. Finally, New York Heart Association (NYHA) class was not available in the MIMIC-IV database. Although this is a limitation, the inclusion of LVEF and other objective covariates provided reasonable adjustment for cardiac function.

## 5 Conclusion

In conclusion, our study demonstrates that the PALBI score serves as an independent prognostic marker for ICU-admitted HF patients. Elevated PALBI scores were significantly linked to higher all-cause mortality at both 30 and 360 days. Additionally, machine learning models incorporating PALBI showed good performance in predicting 30-day mortality. These findings suggest that the PALBI score may be a valuable tool for risk stratification and supporting treatment decisions in the intensive care management of HF.

## Data Availability

The original contributions presented in the study are included in the article/Supplementary Material, further inquiries can be directed to the corresponding authors.
