# Supplementary material for: Association between the platelet-albumin-bilirubin score and all-cause mortality in ICU-admitted heart failure patients: a retrospective cohort analysis and machine learning-based prognostic modeling
Source: Front Cardiovasc Med. 2025 Oct 23;12:1622554. doi: 10.3389/fcvm.2025.1622554 (PMC12589067; doi:10.3389/fcvm.2025.1622554)
Supplement: Supplementary file 1 [file Table1.docx]

**Supplementary materials**

**Supplementary Tables**

**Supplementary Table 1** Missing data rates of clinical variables in ICU patients with heart failure.

| **Variable** | **Missing Rate (%)** |
| --- | --- |
| PTT | 4.69581309 |
| Urine output | 4.48762433 |
| INR | 4.34883183 |
| PT | 4.34883183 |
| Temperature | 2.54452926 |
| Calcium | 1.34166088 |
| SBP | 0.20818876 |
| DBP | 0.20818876 |
| Heart rate | 0.18505667 |
| RR | 0.18505667 |
| SpO2 | 0.18505667 |
| SOFA score | 0.18505667 |
| WBC count | 0.18505667 |
| RDW | 0.18505667 |
| RBC count | 0.11566042 |
| BUN | 0.09252834 |
| Creatinine | 0.04626417 |
| Anion gap | 0.04626417 |
| Sodium | 0.02313208 |
| Potassium | 0.02313208 |
| PTT, partial thromboplastin time; INR, international normalized ratio; PT, prothrombin time; SBP, systolic blood pressure; DBP, diastolic blood pressure; RR, respiratory rate; SpO2, pulse oxygen saturation; SOFA, sequential organ failure assessment; WBC, white blood cell count; RDW, red cell distribution width; RBC, red blood cell count; BUN, blood urea nitrogen. | |

**Supplementary Table 2** Variance inflation factor (VIF) analysis of covariates included in the multivariable Cox regression model.

| Variable | GVIF | Df | GVIF^(1/(2*Df)) |
| --- | --- | --- | --- |
| Gender | 1.063 | 1 | 1.031 |
| AKI | 1.027 | 1 | 1.014 |
| PALBI score | 1.105 | 1 | 1.051 |
| AF | 1.076 | 1 | 1.037 |
| Race | 1.031 | 1 | 1.015 |
| SBP | 1.059 | 1 | 1.029 |
| Myocardial Infarct | 1.046 | 1 | 1.023 |
| Cerebrovascular disease | 1.022 | 1 | 1.011 |
| WBC | 1.027 | 1 | 1.013 |
| Potassium | 1.134 | 1 | 1.065 |
| Creatinine | 1.163 | 1 | 1.078 |
| INR | 1.097 | 1 | 1.048 |
| Beta blockers | 1.049 | 1 | 1.024 |
| LVEF category | 1.057 | 3 | 1.009 |
| PALBI, platelet–albumin–bilirubin score; HR, hazard ratio; CI, confidence interval; SBP, systolic blood pressure; AF, atrial fibrillation; AKI, acute kidney injury; WBC, white blood cell count; INR, international normalized ratio. | | | |

**Supplementary Table 3** Univariate Cox regression analysis of risk factors for all-cause mortality in ICU patients with heart failure.

| **Variables** | ***P*** | **HR (95%CI)** |
| --- | --- | --- |
|  |  |  |
| Gender |  |  |
| Female |  | 1.00 (Reference) |
| Male | 0.617 | 0.97 (0.86 ~ 1.10) |
| Race |  |  |
| Other |  | 1.00 (Reference) |
| White | **0.009** | 0.85 (0.75 ~ 0.96) |
| Age | **<.001** | 1.03 (1.02 ~ 1.03) |
| Heart rate | 0.092 | 1.00 (1.00 ~ 1.01) |
| SBP | **<.001** | 0.99 (0.99 ~ 0.99) |
| DBP | **<.001** | 0.99 (0.99 ~ 0.99) |
| RR | **<.001** | 1.02 (1.01 ~ 1.03) |
| Temperature | **<.001** | 0.74 (0.70 ~ 0.79) |
| SOFA | **<.001** | 1.11 (1.08 ~ 1.13) |
| OASIS | **<.001** | 1.07 (1.06 ~ 1.08) |
| MI |  |  |
| No |  | 1.00 (Reference) |
| Yes | **0.008** | 1.19 (1.05 ~ 1.34) |
| AF |  |  |
| No |  | 1.00 (Reference) |
| Yes | **<.001** | 1.29 (1.15 ~ 1.46) |
| CeVD |  |  |
| No |  | 1.00 (Reference) |
| Yes | **<.001** | 1.46 (1.25 ~ 1.70) |
| COPD |  |  |
| No |  | 1.00 (Reference) |
| Yes | 0.058 | 1.13 (1.00 ~ 1.28) |
| AKI |  |  |
| No |  | 1.00 (Reference) |
| Yes | **<.001** | 2.66 (2.08 ~ 3.40) |
| Diabetes |  |  |
| No |  | 1.00 (Reference) |
| Yes | 0.195 | 1.08 (0.96 ~ 1.23) |
| Hypertension |  |  |
| No |  | 1.00 (Reference) |
| Yes | 0.227 | 1.09 (0.95 ~ 1.27) |
| WBC | **<.001** | 1.01 (1.01 ~ 1.02) |
| RDW | **<.001** | 1.11 (1.08 ~ 1.13) |
| Potassium | **<.001** | 1.18 (1.11 ~ 1.26) |
| Sodium | 0.457 | 1.00 (0.99 ~ 1.01) |
| Calcium | **0.005** | 0.91 (0.85 ~ 0.97) |
| BUN | **<.001** | 1.01 (1.01 ~ 1.01) |
| Creatinine | **<.001** | 1.06 (1.03 ~ 1.09) |
| Aniongap | **<.001** | 1.05 (1.04 ~ 1.06) |
| INR | **<.001** | 1.09 (1.06 ~ 1.13) |
| PT | **<.001** | 1.01 (1.01 ~ 1.01) |
| PTT | **<.001** | 1.01 (1.01 ~ 1.01) |
| Urineoutput | **<.001** | 0.99 (0.99 ~ 0.99) |
| ACEI/ARB |  |  |
| No |  | 1.00 (Reference) |
| Yes | **<.001** | 0.58 (0.51 ~ 0.67) |
| Beta-blockers |  |  |
| No |  | 1.00 (Reference) |
| Yes | **<.001** | 0.47 (0.42 ~ 0.54) |
| Statin |  |  |
| No |  | 1.00 (Reference) |
| Yes | 0.107 | 0.90 (0.80 ~ 1.02) |
| CRRT |  |  |
| No |  | 1.00 (Reference) |
| Yes | **<.001** | 2.84 (2.44 ~ 3.30) |
| SBP, systolic blood pressure; DBP, diastolic blood pressure; RR, respiratory rate; SOFA, sequential organ failure assessment; OASIS, Oxford acute severity of illness score; MI, myocardial infarction; AF, atrial fibrillation; CeVD, cerebrovascular disease; COPD, chronic obstructive pulmonary disease; AKI, acute kidney injury; WBC, white blood cell count; RDW, red cell distribution width; BUN, blood urea nitrogen; INR, international normalized ratio; PT, prothrombin time; PTT, partial thromboplastin time; ACEI, angiotensin-converting enzyme inhibitor; ARB, angiotensin receptor blocker; CRRT, continuous renal replacement therapy. | | |

**Supplementary Figures**

**Supplementary Figure 1**

**
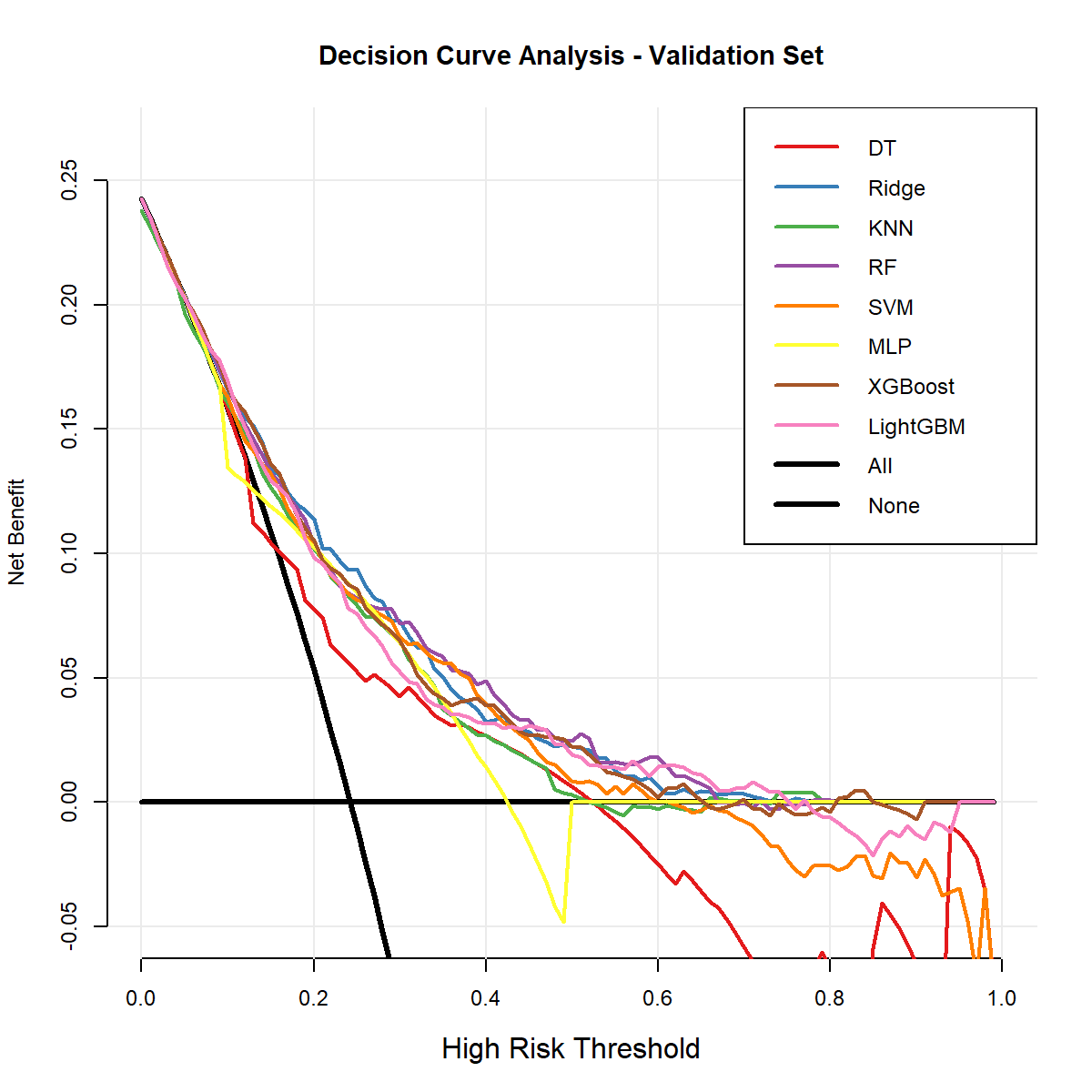
**

**Supplementary Figure 1** Decision curve analysis (DCA) of different machine learning models for predicting all-cause mortality.
